# Challenge-Oriented Organizational Citizenship Behaviors among Nurses: The Influence of Perceived Inclusive Leadership and Organizational Justice in High-Intensity Work Environment

**DOI:** 10.1155/2024/3032694

**Published:** 2024-02-02

**Authors:** Zhen Li, Kefan Chen, Qing Meng, Zhaoli Meng, Yu Zhao, Dehui Li

**Affiliations:** ^1^Department of Otorhinolaryngology-Head & Neck Surgery, West China Hospital of Sichuan University, Chengdu, China; ^2^Department of Audiology and Speech Language Pathology, West China Hospital of Sichuan University, Chengdu, China; ^3^West China School of Nursing, Sichuan University, Chengdu, China; ^4^Department of Strategic and Human Resource Management, Business School, Sichuan University, Chengdu, China

## Abstract

**Aims:**

This study was designed to investigate the impact of inclusive leadership on challenge-oriented citizenship behaviors and examine the mediating role of organizational justice on the relationship between inclusive leadership and challenge-oriented citizenship behaviors among nurses.

**Background:**

Leaders exhibiting an inclusive leadership style have the potential to create a positive work climate and motivate members of the organization. However, the mechanisms by which organizational justice contributes to this process remain to be explored, particularly in terms of how it motivates challenge-oriented organizational citizenship behaviors.

**Method:**

A cross-sectional questionnaire survey was conducted among nurses in China at the end period of the COVID-19 pandemic. A total of 527 registered nurses were enrolled and completed the self-report questionnaires including inclusive leadership scale, organizational justice scale, and challenge-oriented citizenship behavior scale. The hypotheses were tested using hierarchical multiple regression analyses and structural equation modelling.

**Results:**

The results of empirical tests revealed that nurse leader's inclusive leadership had a positive relationship on nurses' challenge-oriented organizational citizenship behaviors (*β* = 0.823, *p* < 0.001) after controlling several demographic covariates. Meanwhile, inclusive leadership was positively linked to organizational justice (*β* = 0.747, *p* < 0.001), and the indirect effect of inclusive leadership on nurses' challenge-oriented citizenship behaviors through organizational justice was statistically significant (*β* = 0.641, *p* < 0.001). Furthermore, the model fit indices were *χ*^2^/*df* = 1.952, RMSEA = 0.043, CFI = 0.979, and TLI = 0.977, indicating that the model had high quality.

**Conclusion:**

This study could help nurse leaders with inclusive leadership style have a better understanding and taking the advantages of the influence mechanism of organizational justice in inspiring nurses' challenge-oriented citizenship behaviors. While nurse managers can inspire challenge-oriented organizational citizenship behaviors through inclusive leadership, they should also emphasize the evaluative and behavioral-shaping effects of organizational justice. Both leadership and organizational justice are essential to motivate challenge-oriented organizational citizenship behaviors and to foster organizational development. Moreover, managers should focus on the process and conditions of implementing organizational justice to ensure fairness within the organization and to create a conducive environment for challenge-oriented organizational citizenship behaviors.

## 1. Introduction

Nurses operate within high-pressure and high-intensity environments, commonly encountering unpredictable circumstances which necessitate effective responses to challenges [1]. Apart from possessing seasoned professional expertise and exceptional clinical skills, nurses also require competent team leadership and organizational support [2]. For instance, during the COVID-19 pandemic, nurses had to carry out extensive medical and public health tasks requiring them to address issues related to complex and stressful circumstances [3], communication [4], work pressure [5], psychological stress [6], and burnout [7]. Consequently, they must frequently restructure and refine their professional skills, working procedures, and management systems, while undertaking extrarole behaviors [8].

Inclusive leadership is often emphasized to motivate nurses to make a greater contribution at work and achieve their full potential. Nonetheless, the practical challenge lies in the complexity of nursing work, which necessitates responses to patient and public health needs based on factors such as the nurse's ability level, patient physical state, and the operational status of the healthcare institution. As a result, the nurse's work is frequently challenging to delimit precisely, and expectations regarding their performance are frequently high. Social exchange theory posits that an individual's behavior is influenced by their perception of justice [9]. In uncertain work environments, nurses possess the drive and demand to exhibit organizational citizenship behaviors; however, this does not necessarily lead to the occurrence of such behaviors, particularly when they are not explicitly defined in formal work duties and can introduce changes and challenges to existing organizational work procedures. This can also lead to relational conflict [10], underscoring the necessity for challenge-oriented organizational citizenship behaviors to have corresponding requirements for the organization's leadership style and atmosphere.

While the interest in inclusive leadership continues to gain traction among scholars, current research predominantly focuses on nurses as research subject to explore the role of inclusive leadership in promoting nurses' psychological perception and behavioral abilities [8]. The inclusive practices of leaders are not only instrumental in shaping employees' personal and group experiences but also the inclusiveness requirements and strategies of an organization [11]. Therefore, the occurrence of certain behavior must be contextualized. Furthermore, the conditions and processes that determine the effectiveness of inclusive leadership, and the factors that facilitate or hinder its role, require extensive exploration and elucidation.

To bridge the research gap, this study intends to investigate the relationship between inclusive leadership and challenge-oriented organizational citizenship behaviors among nurses. Drawing upon the social exchange theory [12], we argue that inclusive leadership facilitates nurses' perception of organizational justice [13], which leads them to feel that they are being treated fairly, whether it is tangible or intangible and consequently enhances their morale, work motivation, loyalty, and stability. Moreover, organizational justice plays a mediating role in stimulating positive challenge-oriented organizational citizenship behaviors while reducing the potential conflicts caused by such behaviors.

## 2. Theory and Hypotheses

### 2.1. Inclusive Leadership

The concept of inclusive leadership was first described by Nembhard and Edmondson [14] as “words and deeds by a leader or leaders that indicate an invitation and appreciation for others' contribution.” Carmeli et al. [15] further defined it as the core of relational leadership, a leadership style that exhibits openness, availability, and accessibility in interactions with subordinates. Inclusive leadership implies that leaders not only exhibit the characteristics of recognizing employee's differences [16], collaborating during decision-making [17], promoting creativity through cognitive mechanism [18], and cultivating the ability of the employee to learn and work as a catalyst for the achievement of organizational goals [19] but also means that the entire organizational team is concerned about new opportunities [20], prepares for organizational change [21], and supports for organizational structure and culture [22], thus having a tremendous impact on the creative process of the organization [15, 23].

### 2.2. Inclusive Leadership and Challenge-Oriented Citizenship Behaviors

Challenge-oriented organizational citizenship behaviors are a kind of extrarole behaviors [24]. It refers to the active and intentional participation of individuals in organizational development and performance improvement, by putting forward creative ideas or encouraging change efforts related to working methods, policies, and processes in promotive forms [25]. Meanwhile, it is also a kind of proorganizational behavior “that can neither be enforced on the basis of formal role obligations nor elicited by contractual guarantees of recompense” [26, 27].

Interestingly, challenge-oriented organizational citizenship behaviors have a double-edged sword effect. On the one hand, challenge-oriented behaviors may undermine task performance and relationships with team colleagues because these suggestions require changes to the status quo, yet change means that people affected by these changes have to adapt to something new and often involve setbacks and failure [28]. On the other hand, the most prominent form of facilitation of challenge-oriented citizenship behaviors is employee voice, which is characterized by constructive suggestions for the benefit of the organization [26, 29].

Inclusive leadership may have positive impact on challenge-oriented organizational citizenship behaviors in three ways. First, inclusive leadership provides space and emotional ties to challenge-oriented organizational citizenship behaviors [30, 31]. The supportive behaviors of inclusive leaders result in employees feeling that they are treated well and with respect, which motivates the receiving party attempts to reciprocate with something equally valuable [32]. Second, inclusive leadership provides positive guidance for challenge-oriented organizational citizenship behaviors, thus avoiding the risk of the prohibitive forms of challenge-oriented behaviors in damaging relationships with others [25, 33]. The effective behaviors of inclusive leadership are conducive to resolving the negative impact of relationship conflicts [34]. Finally, when employees observe that leaders have characteristics of inclusive leadership, they are more inclined to express their concerns and voices to the leader, and communication with good response and reciprocity further strengthens the possibilities of trust-building and problem-solving [35], which makes inclusive leadership as a precondition to challenge-oriented organizational citizenship behaviors. Based on this reasoning, we propose the following hypothesis:

 
*H1*. Inclusive leadership of nurse managers has a positive effect on nurses' challenge-oriented citizenship behaviors.

### 2.3. The Mediating Role of Organizational Justice

Organizational justice is based on Equity Theory [36] and can be used to describe and explain employees' perceptions of fairness and honesty of the treatment they received [13], including procedural justice, distributive justice, and interactional justice. First, procedural justice refers to the fairness of rules and procedures and is fostered “when authorities provide employees with input into key decisions and when authorities utilize procedures that are consistent, accurate, unbiased, correctable, representative of group concerns, and ethical” [37]. Second, distributive justice refers to the perceived fairness of the outcomes that individuals receive from organizations and is fostered when outcomes conform to implicit distributive norms, such as equity or equality [38]. Third, interactional justice refers to the individuals' perception and judgment on the quality of interpersonal treatment received during the execution of a procedure [39], including informational justice (the provision of adequate information and social accounts) and interpersonal justice (the dignity and respect that one receives) [40, 41].

Inclusive leadership has an impact on challenge-oriented organizational citizenship behaviors via organizational justice. First, the open and supportive behaviors of inclusive leaders can improve procedural justice in organizations, as employees have the perception that their voices are heard by managers and that they are included in the decision-making process [42]. In terms of procedural justice, the involvement of team members in the decision-making process is of great significance, which determines and achieves an organizational decision-making process that is consistent, accurate, unbiased, correctable, and ethical [37]. At the same time, it shapes member's assessment of the authority's trustworthiness [43] and reflects employees' concerns about the evaluation of procedural justice and the participation in challenge-oriented organizational citizenship behaviors [44]. Second, the perceived organizational justice makes employees feel trust in the process and distribution of outcomes of their work and therefore inspires their extrarole behaviors that contribute to effective organizational functioning [43]. In particular when employees feel that the distribution of rewards based on their work input is fair, that the process of allocating resources and rewards is transparent, and that they are treated with mutual respect, these feelings create a sense of obligation to give back to the organization [45]. Third, under inclusive leadership, employees tend to feel respected, which indicates an increased level of interactional justice in the organization [46]. The open behaviors in discussing with employees reinforce the perception of interactional justice, as employees are more likely to feel that they have received all the necessary information and that well-developed interpersonal justice allows them not to be disturbed by interpersonal relationships, which in turn motivates them to change organizational rules or policies that are nonproductive or counterproductive, which also makes it possible to mitigate the potential conflicts in challenge-oriented organizational citizenship behaviors and stimulate positive effects. In recent years, studies on healthcare teams have revealed that organizational justice has effects on work engagement and nursing care quality [47], reducing turnover intention [48], and motivating the performance and productivity of nurses [49]. Therefore, we argue that inclusive leadership has a positive contribution to the perception of organizational justice, and meanwhile, organizational justice is a connecting mechanism in the relationship between inclusive leadership and challenge-oriented citizenship behaviors. Thus, the following hypotheses were developed:

 
*H2*. Inclusive leadership of nurse managers has a positive effect on organizational justice
 
*H3*. Organizational justice mediates the relationship between inclusive leadership of nurse managers and nurses' challenge-oriented citizenship behaviors

## 3. Methods

### 3.1. Research Design and Participants

We conducted a cross-sectional survey to examine the relationship between inclusive leadership and challenge-oriented citizenship behaviors and the moderating role of organizational justice among nurses in China at the end period of the COVID-19 pandemic. The study followed the Strengthening the Reporting of Observational Studies in Epidemiology (STROBE) guidelines. Convenient sampling was used to recruit the nurses who were registered full-time nurses and had more than six months of tenure in their current hospital and were willing to participate in this study. The minimum sample size was estimated by the maximum one of following two methods [50, 51]: (1) using PASS (Version 20.0, https://www.ncss.com/software/pass) with 90% power, alpha of 0.05, a medium *f*^2^ effect size of 0.15 and 34 predictors, and assuming an attrition rate of 20%, 303 samples were estimated; (2) multiplying the number of items of the instruments by ten with assuming an attrition rate of 20%, (9 + 20 + 5) × 10 × (1 + 20%) = 408 samples were estimated. In general, a sample size of approximately more than 400 would provide sufficient support for this study.

### 3.2. Instruments

#### 3.2.1. Inclusive Leadership Scale

The inclusive leadership scale was used to measure the nurse manager's inclusive leadership style [15]. The scale consists of nine items based on three dimensions including openness (three items), availability (four items), and accessibility (two items). A sample item is, “The manager is open to discuss the desired goals and new ways to achieve them.” Each item is scored on a 7-point Likert scale, ranking from one (strongly disagree) to seven (strongly agree). A total inclusive leadership score was obtained by averaging the three dimensions, where the higher score indicated a higher level of nurses' perception of their leader's inclusive leadership.

#### 3.2.2. Organizational Justice Scale

The organizational justice at work among nurses was measured using the scale developed by Colquitt [40]. It consists of twenty items divided into four subscales: procedural justice, distributive justice, interpersonal justice, and informational justice. The first subscale is procedural justice, with 7-item statements that ask nurses to consider the procedures their supervisor uses to make decisions about evaluations, promotions, and rewords. The second subscale, distributive justice, has 4-item statements that ask nurses to consider how they were treated by their supervisor during the implementation of procedures. The third subscale is interpersonal justice, with 4-item statements that ask nurses to consider the outcomes they received from their supervisor, including their evaluations, promotions, and rewards. Finally, the fourth subscale is informational justice, with 5-item statements that ask nurses to consider their perceived adequacy of explanations from their supervisor during the implementation of procedures. The items are scored on a 7-point Likert scale ranging from one (strongly disagree) to seven (strongly agree), with the items being answered based on how often the nurses encounter each statement. The total score was created from the average of all items, with higher scores representative of greater organizational justice.

#### 3.2.3. Challenge-Oriented Organizational Citizenship Behaviors Scale

A 5-item measure validated by Mackenzie et al. [25] was used to measure challenge-oriented citizenship behaviors, which was drawn from the original scale developed by Van Dyne and LePine [24]. Each item is scored on a 7-point Likert scale, ranking from one (strongly disagree) to seven (strongly agree). A total challenge-oriented citizenship behaviors score was obtained by averaging all items, where the higher score indicated a higher level of nurses' challenge-oriented citizenship behaviors.

### 3.3. Data Collection

For the convenience of the participants and to expand the scope of the survey, the questionnaire of this study was conducted both offline and online. In the offline survey, printed questionnaires were sent to participants by the authors and recorded into the database after the questionnaires were completed. The online questionnaire was set up using a widely used electronic questionnaire collection platform named Questionnaire Star (https://www.wjx.cn). The internet link to the electronic questionnaire was first sent through the authors to nurses in different cities and hospitals, and they were invited to continue sharing the questionnaire with nurses in other regions and hospitals they knew. A cash coupon of RMB 5 for each offline participant and a random electronic cash coupon of RMB 5 to RMB 10 for each online participant were provided in this study, which was implemented through the Questionnaire Star platform. In order to ensure the quality of the questionnaire design and responses, on the one hand, we designed reverse repetition questions in the questionnaire to check whether the participants answered the questionnaire carefully. On the other hand, we invited 30 nurses to conduct a pilot study for evaluating the clarity and time needed to fill out the questionnaires. The questionnaire took approximately 8–15 minutes to complete, and with reference to the previous literature [52], we removed the samples with response time less than 3 minutes and no variation in all response items; for example, all items were selected as “strongly disagree.” The survey was available from October 10, 2022, to February 10, 2023, and 527 valid questionnaires were obtained for this study.

### 3.4. Analysis

Statistical analyses were conducted using IBM SPSS (Version 26) and AMOS (Version 26). Descriptive statistics, which included frequency and proportions, were employed to summarize the demographic characteristics of age, gender, education, marital status, years of experience in nursing, job title, and the experience in frontline work against COVID-19 in the past three years.

Hierarchical multiple regression analysis was performed to estimate the direct effect of inclusive leadership on challenge-oriented citizenship behaviors after controlling other variables. Structural equation modelling (SEM) with multiple indices criteria was conducted to determine the influence of inclusive leadership and organizational justice on challenge-oriented citizenship behaviors. In addition, to evaluate the measurement and factor structure of the study variables, confirmatory factor analysis (CFA), including KMO, Bartlett test of sphericity, and the average variance extracted (AVE) were performed to ensure the validity of the study construct, and Cronbach's alpha and composite reliability (CR) were estimated to ensure the reliability of the items of the scale used in this study [51, 53, 54].

### 3.5. Ethical Considerations

After scrutinizing all the necessary documents, including design of this research, written questionnaire, academic review opinions, subject informed consent, researchers' biographies, and description of source of foundations, the Biomedical Ethics Committee of the West China School of Medicine and the West China Hospital of Sichuan University granted permission to conduct this study (#2022-1479; approved on 9 September 2022). The first page of the questionnaire stated the purpose of the study, while emphasizing the voluntariness, anonymity, and confidentiality of the answers, and the participants began to answer the questionnaire survey after agreeing to the above contents.

## 4. Results

### 4.1. Demographic Profile of Participants

It can be seen in Table 1 that predominant participants had age between 26 and 40 years (59.8%), were female (85.39%), had a bachelor's degree (65.84%), and had married (71.35%). From the distribution characteristics of the participants, their years of work experience were relatively evenly distributed, ranging from 24.10% to 25.43% in the four levels classified in this study. In terms of the participants' job titles, 87.10% of the participants were nurse or senior nurse, 11.01% were leader of nurse team, and 1.90% of the participants were nurse supervisor or above, and these distribution characteristics were consistent with our estimation of the overall pyramid structure. In addition, more than half of the participants (55.60%) were involved in frontline work against COVID-19 in the past three years.

### 4.2. Reliability and Validity

Construct reliability was analyzed, Cronbach's alpha and CR, to verify the internal consistency of the instruments in the study (Table 2). Cronbach's alpha should be preferably over the recommended level of 0.7. In our study, Cronbach's alpha values ranged from 0.911 to 0.948 for all first-order scales, showing a high degree of internal consistency within the scales. In addition, the CR values excessed the recommended level of 0.7 for all dimensions in our study scales, indicating a high degree of convergence and internal consistency [51].

Meanwhile, construct validity was verified the degree to which the instrument accurately represents the concept is defined in the study. First, the Kaiser–Meyer–Olkin (KMO) test and Bartlett test of sphericity were used to examine sampling adequacy. The KMO value should be at least 0.60, and the Bartlett test of sphericity should be statistically significant at *p* < 0.05. The results revealed that the KMO and Bartlett test of sphericity values were 0.965 (*p* < 0.001) for the inclusive leadership scale, 0.968 (*p* < 0.001) for the organizational justice scale, and 0.913 (*p* < 0.001) for the scale of the challenge-oriented citizenship behaviors. Second, the factor loading of all constructs employed in this study exceeds the threshold value of 0.7, indicating a high degree of correspondence between the variable and the factor. Furthermore, the average variance extracted (AVE) values were higher than 0.50 thresholds for all dimensions of the study variables, indicating that the convergent validity is well-supported.

### 4.3. Hypothesis Test Results

Table 3 presents the results of the hierarchical multiple regression analysis to test the effect of inclusive leadership on nurses' challenge-oriented citizenship behaviors. After controlling the demographic variables in Model 1 (age, gender, educational background, marital status, years of experience, job position, and the work experience in COVID-19) as covariates, the inclusive leadership statistically significantly enhances nurses' challenge-oriented citizenship behaviors (*β* = 0.823, *p* < 0.001) in Model 2, with 50.6% increasing of adjusted *R*^2^. Thus, H1 was supported.

**Figure 1:**
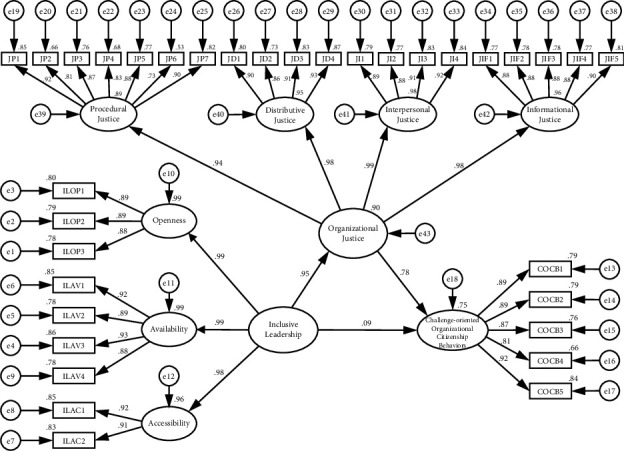
Structural equation model results.

Table 4 and Figure 1 illustrate that inclusive leadership was positively associated with organizational justice (*β* = 858, *p* < 0.001), which supported H2, and organizational justice was positively associated with nurses' challenge-oriented citizenship behaviors (*β* = 0.747, *p* < 0.001). In addition, after incorporating organizational justice into the model, the direct effect of inclusive leadership on nurses' challenge-oriented citizenship behaviors was no longer statistically significant (*β* = 0.075, *p* > 0.05). In the bootstrap test with AMOS (Version 26), the results of 5000 bootstrapping resamples revealed that the estimated coefficient of the indirect effect of inclusive leadership on nurses' challenge-oriented citizenship behaviors was statistically significant (*β* = 0.641, *p* < 0.001). In sum, these empirical results indicated that organizational justice acts as a mediator in the relationship between inclusive leadership and nurses' challenge-oriented citizenship behaviors. Furthermore, the model fit indices were *χ*^2^/*df*  = 1.952, RMSEA = 0.043, CFI = 0.979, and TLI = 0.977, indicating that the model had high quality. Thus, H3 was supported.

## 5. Discussion

Nurses operate in demanding and intense work environments where the leadership style demonstrated by leaders is considered crucial in facilitating effective nursing practices. In our research, we argue that inclusive leadership does not automatically result in challenge-oriented organizational citizenship behaviors, particularly those that lack clear definition within formal job responsibilities and tend to disrupt and question existing organizational practices. By integrating social exchange theory [12] and organizational justice theory [13] into our investigation, we have discovered that inclusive leadership positively influences nurses' inclination towards engaging in challenge-oriented organizational citizenship behaviors, with organizational justice serving as a mediating mechanism.

This paper contributes theoretically and practically in several ways. First, previous research has highlighted the crucial role of effective managerial support in fostering organizational citizenship behaviors. Team members can assess their level of engagement in such behaviors by observing the conduct of inclusive leaders. Inclusive leaders who exhibit supportive and helpful behaviors, such as being readily available to assist others, are perceived as trustworthy by team members. As a result, team members are more likely to exhibit extrarole behaviors towards their colleagues. The supportive behaviors of inclusive leaders contribute to the development of trust among team members. When inclusive leaders demonstrate tolerance towards suggestions and mistakes, they create an open and autonomous organizational environment that encourages active employee participation, proposal of solutions, and surpassing of organizational goals. However, previous research has largely overlooked the significance of procedural safeguards and incentives, particularly in challenging work environments like nursing. In other words, although a manager's leadership style may mitigate the negative effects of job-related friction and conflict, it does not guarantee the occurrence of positive extrarole behaviors among team members. Our research emphasizes that, drawing from social exchange theory, the supportive behavior of inclusive leaders fosters a perception among nurses that they are treated favorably, thereby motivating them to reciprocate towards the leader and the organization. Simultaneously, based on the establishment of organizational justice, this process provides safeguards for the behavior of organizational members and offers incentives for compatibility.

Second, organizational justice plays a crucial role as a motivator and mediator in this process. Existing literature has highlighted that when employees perceive fairness from their supervisors or the organization, they are more likely to engage in organizational citizenship behaviors. In our study, we argue that organizational justice influences challenge-oriented organizational citizenship behaviors for three primary reasons. The first fundamental reason is that a high level of perceived organizational justice fosters employee satisfaction with both the work process and the organization, thereby motivating them to actively participate in extrarole behaviors [24]. The second reason is that organizational justice cultivates trust in the organization and its operational mechanisms [55], thereby reducing perceived anxiety and threat associated with engaging in challenge-oriented organizational citizenship behaviors. Lastly, inclusive leadership and organizational justice operate synergistically. Previous research has predominantly focused on the behavior-driven aspects of inclusive leadership, neglecting the evaluation mechanisms that influence the occurrence of challenging behaviors within organizations. Our findings suggest that fair evaluation mechanisms and confidence in their effectiveness lead organizational members to believe that their creative ideas and challenge-oriented organizational citizenship behaviors, aimed at enhancing organizational performance, will be appropriately recognized. This insight enhances our understanding of the effectiveness of inclusive leadership and organizational justice.

In addition, this study provides guidance to nurse management leaders and nurses in their work practices. On the one hand, inclusive leadership respects and recognizes the commitment and abilities of subordinates, cares for their needs and work status, and reinforces the social exchange of material and emotional relationships, leading to more positive work attitudes and innovative practices based on well-intentioned starting points. On the other hand, inclusive leadership implies a more tolerant attitude towards behaviors that may drive organizational change, which obviously have the potential to cause friction and conflict in the organization; however, organizational justice means that these behaviors are evaluated objectively and appropriately, thus creating a positive motivational effect on the functioning of the organization, which also implies that the leader becomes a facilitator rather than a controller [56]. In sum, how to promote organizational justice and how to motivate challenge-oriented organizational citizenship behaviors is what each member of the organization needs to contemplate, both as an effective working tool in the practice of nurse management, which in many cases is already a necessity, and as the ultimate purpose of achieving iterative renewal of the organization itself.

## 6. Limitations and Future Research

We recognize that this study has some limitations, in that they also offer directions that could be investigated in the future. First, the cross-sectional study design employed in the current study restricts the ability to establish causal relationships. Consequently, future studies should consider both longitudinal and experimental study designs. Second, the study results are based on self-reported data, which may be subject to response bias and subjectivity. Therefore, the findings are only applicable to the sample and responders of the study and cannot be generalized to all contexts, particularly those with unstable organization and structure. Finally, more about individuals, organizations, and the relationships between them and nurse-specific contexts needs to be investigated in future research. On the one hand, as the results of this study, inclusive leadership promotes organizational justice and motivates challenge-oriented organizational citizenship behaviors, then under conditions of such motivational compatibility between individuals and organizations, Person-Organization Fit can be enhanced [57], especially considering the level of compassionate behaviors of the nurses when serving patients, which may not only change the underlying contexts and thresholds at which inclusive leadership functions, but Person-Organization Fit and organizational justice may also complementarily motivate the extrarole behaviors of nurses. On the other hand, in view of the characteristics of nurses' work, they often need to pay more attention to the needs of others for help, which include both physical and psychological, then positive thinking plays an important role as a positive personal resource, which can help nurses self-regulate their negative emotions into a more positive direction, so that they can achieve a higher level of positive emotions and psychological flexibility [58], especially in the context of high-intensity nursing care, leading them to engage in more behaviors beyond their own self-interest.

## 7. Conclusion

The current study provides additional evidence of the necessity of inclusive leadership in building organizational justice and inspiring nurses' challenge-oriented organizational citizenship behaviors. At the same time, this study reveals and validates the mediating role of organizational justice in the mechanism of inclusive leadership on challenge-oriented organizational citizenship behaviors. Nurse leaders can motivate such extrarole behaviors by consciously establishing organizational justice, which also means that organizational justice has the dual property of being the purpose and the effective methods of nurse management.

## Figures and Tables

**Table 1:** Demographic profile of the participants (*n* = 527).

Characteristics	Frequency (*f*)	Percentage (%)
*Age (years)*		
18–25	124	23.53
26–30	113	21.44
31–40	202	38.33
>40	88	16.70

*Gender*		
Female	450	85.39
Male	76	14.42
Non-disclosure	1	0.19

*Education*		
Diploma or lower	132	25.05
Bachelor	347	65.84
Master	38	7.21
PhD	10	1.90

*Marital status*		
Unmarried	144	27.32
Married	376	71.35
Divorced or other	7	1.33

*Years of experience*		
1–5 years	134	25.43
6–10 years	133	25.24
11–15 years	127	24.10
More than 15 years	133	25.24

*Job title*		
Nurse and senior nurse	459	87.10
Leader of nurse team	58	11.01
Nurse supervisor or above	10	1.90

*Frontline nurses in COVID-19*		
Yes	293	55.60
No	234	44.40

**Table 2:** Test results of reliability and validity.

Scale	No. of items	Mean (SD)	KMO	Bartlett test	Loading range	*α*	CR	AVE
Inclusive leadership	**9**	**5.20 (1.59)**	**0.965**	**<0.001**	**0.873–0.926**			
Openness	3	5.22 (1.61)	0.760	<0.001		0.917	0.918	0.789
Availability	4	5.17 (1.58)	0.866	<0.001		0.947	0.948	0.820
Accessibility	2	5.24 (1.61)	0.500	<0.001		0.911	0.911	0.837
Organizational justice	**20**	**5.25 (1.55)**	**0.968**	**<0.001**	**0.742–0.935**			
Procedural justice	7	5.15 (1.53)	0.946	<0.001		0.948	0.948	0.724
Distributive justice	4	5.32 (1.55)	0.859	<0.001		0.943	0.944	0.808
Interpersonal justice	4	5.34 (1.59)	0.870	<0.001		0.944	0.844	0.810
Informational justice	5	5.25 (1.54)	0.915	<0.001		0.947	0.948	0.784
Challenge-oriented citizenship behaviors	**5**	**5.26 (1.45)**	**0.913**	**<0.001**	**0.809–0.919**	0.943	0.943	0.768

SD, standard deviation; CR, composite reliability; AVE, average variance extracted. The rows with regular font values are the first order and the bold values represent the second order for inclusive leadership and organizational justice.

**Table 3:** Multiple linear regression analysis of inclusive leadership on challenge-oriented citizenship behaviors.

Variables	Challenge-oriented citizenship behaviors							
	Model 1				Model 2			
	*β*	Std. err	*t*	*p*	*β*	Std. err	*t*	*p*
Main effect variables								
Inclusive leadership					0.823	0.030	27.46	<0.001
Control variables								
Age (years)								
18–25	−1.344	0.425	−3.17	0.002	−0.269	0.273	−0.98	0.325
26–30	−0.879	0.378	−2.33	0.020	−0.180	0.242	−0.75	0.456
31–40	−0.349	0.284	−1.23	0.219	0.108	0.181	0.60	0.550
>40	(omitted)				(omitted)			
Gender								
Female	0.881	0.202	4.36	<0.001	0.189	0.131	1.44	0.150
Male	(omitted)				(omitted)			
Education								
Diploma or lower	−0.521	0.494	−1.05	0.292	−0.400	0.314	−1.27	0.203
Bachelor	−0.253	0.477	−0.53	0.596	−0.242	0.303	−0.80	0.425
Master	0.726	0.532	1.37	0.173	0.380	0.339	1.12	0.262
PhD	(omitted)				(omitted)			
Marital status								
Unmarried	−0.260	0.595	−0.44	0.663	0.202	0.378	0.53	0.593
Married	0.185	0.624	0.30	0.767	0.676	0.397	1.70	0.089
Divorced or other	(omitted)				(omitted)			
Years of experience								
1–5 years	0.603	0.395	1.53	0.127	−0.239	0.253	−0.94	0.346
6–10 years	−0.445	0.318	−1.40	0.163	−0.329	0.202	−1.63	0.104
11–15 years	−0.525	0.265	−1.98	0.049	−0.486	0.169	−2.88	0.004
More than 15 years	(omitted)				(omitted)			
Job title								
Nurse and senior nurse	−0.779	0.480	−1.62	0.105	−0.520	0.305	−1.70	0.089
Leader of nurse team	−0.576	0.515	−1.12	0.264	−0.785	0.328	−2.40	0.017
Nurse supervisor or above					(omitted)			
Frontline in COVID-19								
Yes	0.191	0.134	1.43	0.154	0.038	0.085	0.45	0.653
No	(omitted)				(omitted)			
_cons	7.323	0.900	8.14	<0.001	2.638	0.597	4.42	<0.001
Adjusted *R*^2^	0.150				0.657			
Δ*R*^2^					0.506			

**Table 4:** Mediation effect of organizational justice between inclusive leadership and challenge-oriented citizenship behaviors.

Structural path	*β*	Std. err	*t*	*p*	95% conf. interval	
					Lower	Upper
*Direct effect*						
Inclusive leadership ⟶ organizational justice	0.858	0.032	26.813	<0.001	0.793	0.920
Organizational justice ⟶ challenge-oriented citizenship behaviors	0.747	0.148	5.047	<0.001	0.456	1.034
Inclusive leadership ⟶ challenge-oriented citizenship behaviors	0.075	0.131	0.573	0.560	−0.172	0.340

*Indirect effect*						
Inclusive leadership ⟶ challenge-oriented citizenship behaviors	0.641	0.123	5.211	<0.001	0.397	0.878

*Total effect*						
Inclusive leadership ⟶ challenge-oriented citizenship behaviors	0.715	0.034	21.03	<0.001	0.644	0.780

*Note.* Estimate based on 5000 bootstrap resamples.

## Data Availability

The data that support the findings of this study are available from the corresponding author upon reasonable request. The data are not publicly available because of privacy or ethical restrictions.
